# Chemerin suppresses hepatocellular carcinoma metastasis through CMKLR1-PTEN-Akt axis

**DOI:** 10.1038/s41416-018-0077-y

**Published:** 2018-05-02

**Authors:** Jing-Jing Li, Hong-Kun Yin, Dong-Xian Guan, Jiang-Sha Zhao, Yu-Xiong Feng, Yue-Zhen Deng, Xiang Wang, Nan Li, Xiao-Fan Wang, Shu-Qun Cheng, Ying Bao, Dong Xie

**Affiliations:** 1 Key Laboratory of Nutrition, Metabolism and Food Safety, Shanghai Institutes for Biological Sciences, Chinese Academy of Sciences, 320 Yueyang Road, Shanghai, 200031 China; 2 Department of Surgery, First People’s Hospital Affiliated, Huzhou University, Huzhou, 313000 China; 3 Eastern Hepatobililary Surgery Hospital, Second Military Medical University, 225 Changhai Road, Shanghai, 200433 China; 4 Department of Pharmacology and Cancer Biology, Duke University Medical Center, Durham, NC 27710 USA; 5 School of Life Science and Technology, ShanghaiTech University, 393 Middle Huaxia Road, Shanghai, 201210 China

**Keywords:** Metastasis, Cell migration

## Abstract

**Background:**

Chemerin, a known chemoattractant, participates in multiple biological events. However, its role in cancer remains largely unknown.

**Methods:**

Chemerin expression was evaluated by real-time PCR, western blot and immunohistochemistry. Forced expression, RNAi, immunoprecipitation, etc. were used in function and mechanism study. Mouse models of extrahepatic and intrahepatic metastasis were employed to evaluate the therapeutic potential of chemerin.

**Results:**

Chemerin expression was significantly downregulated in hepatocellular carcinoma, and associated with poor prognosis of HCC patients. Forced expression of chemerin inhibited in vitro migration, invasion and in vivo metastasis of HCC cells. Administration of chemerin effectively suppressed extrahepatic and intrahepatic metastases of HCC cells, resulting in prolonged survival of tumour-bearing nude mice. Chemerin upregulated expression and phosphatase activity of PTEN by interfering with PTEN–CMKLR1 interaction, leading to weakened ubiquitination of PTEN and decreased p-Akt (Ser473) level, which was responsible for suppressed migration, invasion and metastasis of HCC cells. Positive correlation between chemerin and PTEN, and reverse correlation between chemerin and p-Akt (Ser473) were also observed in HCC clinical samples and intrahepatic mouse model in vivo.

**Conclusions:**

Our study has revealed the suppressive role and therapeutic potential of chemerin in HCC metastasis, providing both a prognostic marker and drug candidate for HCC.

## Introduction

Worldwide, hepatocellular carcinoma (HCC) is the fifth most common cancer and a leading cause of cancer-related mortality, with an estimated 782,000 new cases and 745,000 deaths in the year 2012.^1^ Though surgery remains the most effective therapeutic approach, the majority of patients undergoing partial hepatectomy will, nevertheless, develop intrahepatic or distant metastases.^2^ Likewise, in about 50% of the patients who die within 5 years after liver transplantation for HCC, intra- and extrahepatic recurrences contributed to their death.^3^ Therefore, it is important to reveal the mechanism of HCC metastasis and develop more effective treatment strategies to prevent HCC metastasis.

Chemerin was originally identified as the product of a gene upregulated by the RAR β/γ-selective anti-psoriatic synthetic retinoid tazarotene.^4^ It was later discovered to be the natural ligand of the orphan G protein-coupled receptor (GPCR) chemokine-like receptor 1 (CMKLR1),^5^ which was mainly expressed in immune cells, including plasmacytoid dendritic cells (pDCs), tissue macrophages^6^ and natural killer cells.^7^ Chemerin was soon recognised as a chemoattractant that promotes the recruitment of immune cells to lymphoid organs and sites of tissue injury. Subsequent studies noted its involvement in numerous biological processes, including adipocyte differentiation, metabolic syndrome and cardiovascular disease. Chemerin suppresses melanoma by recruiting natural killer cell antitumour defense,^8^ however, the role of chemerin other than chemoattractant in cancer remains largely unexplored.

In this study, we reported decreased expression of chemerin in HCC, and its level could serve as an independent risk factor for HCC. We further characterised chemerin as a metastasis suppressor in HCC, which inhibited cell migration and invasion in vitro and metastasis in vivo. This occurred through negative regulation of Akt via CMKLR1-PTEN axis. Administration of chemerin effectively suppressed extrahepatic and intrahepatic metastases of HCC cells in nude mice, prolonged their survival and hindered weight loss. In summary, our study has revealed the novel function and underlying mechanisms of chemerin in HCC, providing a prognostic marker and therapeutic candidate for this malignancy.

## Materials and methods

### Reagents

Anti-human chemerin antibody and recombinant human chemerin, R&D; Anti-chemerin antibody for immunohistochemistry, Phoenix Pharmaceuticals; Anti-PTEN (mouse mAb), anti-ChemR23, anti-Ub and anti-β-actin antibody, Santa Cruz Biotechnology; Anti-Akt, p-Akt (Thr308), p-Akt (Ser473), phospho-GSK3beta (Ser9), PTEN (rabbit mAb) and p-PTEN (Ser380/Thr382/383) antibodies, Cell Signaling Technology; Anti-HA antibody, Sigma; Myeloperoxidase (MPO) antibody, Abcam; Anti-CMKLR1 antibody, Bioworld Technology Inc; Anti-MMP1 antibody, Proteintech Group; Anti-GST antibody, Sangon Biotech (Shanghai); Rat anti-mouse CD68, AbD Serotect Inc; DeadEnd™ Fluorometric TUNEL System, Promega.

### Animals

Six-week-old male BALB/c mice were housed under standard conditions. The animal protocols were done in agreement with SIBS Guide for the Care and Use of Laboratory Animals and approved by Animal Care and Use Committee, Shanghai Institutes for Biological Sciences.

### Tissue samples and tissue microarray analysis

HCC tissue samples and paired cancer-adjacent normal tissues were obtained from Eastern Hepatobiliary Surgery Hospital, after the written informed consent was obtained from the patients. TMA1 is constructed from 320 paraffin-embedded primary HCC tissues, 60 paired non-tumour liver tissues, and 33 corresponding portal vein thrombus tumour tissues. TMA2 is composed of 222 pairs of HCC tissues and paired normal liver tissues. Two experienced pathologists confirmed the pathological diagnosis in each case before the tissue arrays were constructed. The clinical stage was determined according to the TNM (WHO criteria).

TMA1 was immunostained with anti-human chemerin, PTEN, p-Akt (Ser473) and CD68 antibodies, and TMA2 was only immunostained with chemerin. Two experienced pathologists evaluated the immunoreactivity and histological appearance of all tissue samples in the microarray. The staining intensity of tumour cells was scored on a scale of 0–3, with 0 being no staining, 1 as weak intensity, 2 as moderate intensity and 3 as strongest intensity.

### Western blot analysis

Cultured cells or tissues were lysed in RIPA buffer for 15 min on ice. Cell lysates were clarified by centrifugation (14,000 r.p.m., 15 min), and protein concentrations were determined using Bradford Reagent (Bio-Rad). The lysates were separated on 10%/12% SDS-PAGE, and blots were immunoblotted with indicated primary antibodies and the corresponding horseradish peroxidase-conjugated secondary antibodies. All immunoblots were visualised by ECL (Pierce). Intensity of the blots was quantified by Image J.

### Immunohistochemistry

The collected tumours or liver tissues were fixed in 4% formaldehyde solution in PBS, and then embedded in paraffin. Five micrometer thick sections were cut from paraffin-embedded tissue blocks, deparaffinized and rehydrated in dimethylbenzene and ethanol, then subjected to antigen retrieval. The endogenous peroxidase activity was blocked using 0.3% hydrogen peroxide in methanol for 30 min, then the sections were blocked with 3% BSA and 5% NGS in PBS for 1 h at 37 °C, followed by incubation with primary antibodies at 4 °C overnight. After washing with PBS three times on the second day, the corresponding secondary antibodies were applied, and the samples were further incubated at 37 °C for 1 h. Finally, the slides were visualised with DAB staining.

### In vivo metastasis assay using intrahepatic injection model

Intrahepatic injection was performed as described by Tada et al.,^9^ and a total of 5.0 × 10^5^ cells mixed with matrigel at 1:1 ratio (volume) were injected into the left hepatic lobes of nude mice. The injected luciferase-labelled HCC cells were confirmed 3 days after the operation using the living Image system (Xenogen). This intrahepatic injection model consistently yielded detectable tumours in 95–100% of animals.

### Statistical analysis

Statistical analysis was performed by SPSS software, version 13.0 (SPSS, Inc., Chicago, IL, USA). The data distribution was examined using the Kolmogorov–Smirnov test, and the non-normally distributed data were analysed using Mann–Whitney test. Homogeneity of the variance was tested using Levene’s Test. If the variances were homogeneous, the data were analysed by two-tailed unpaired *t* test. For data sets with nonhomogeneous variances, two-tailed unpaired *t* test with Welch correction was applied. Relationship between chemeirn expression and clinical characteristics, p-Akt (Ser473), PTEN expression and macrophages were analysed by *Χ*^2^ test. The survival curves were calculated using the Kaplan–Meier method, and the differences were assessed by a log-rank test. Univariate and multivariate Cox proportional hazards models were used to investigate the association between survival time and patient characteristics. The criterion for significance was *p* < 0.05 for all comparisons.

## Results

### Chemerin is downregulated in HCC and can serve as an independent risk factor for survival

To investigate the mechanism of HCC metastasis, we performed microarray analysis using a metastatic HCC cell line MHCC97 (P), and three MHCC97-derived subclones L, H and M with gradually increased metastatic capabilities.^10,11^ The expression of genes whose values of the three ratios rose gradationally and significantly (L/P < H/P < M/P) were defined as metastasis promoters in HCC. Vice versa, the genes were defined as metastasis suppressors if the ratios decreased (L/P > H/P > M/P), such as chemerin (Supplementary Figure S1A).

**Fig. 1:**
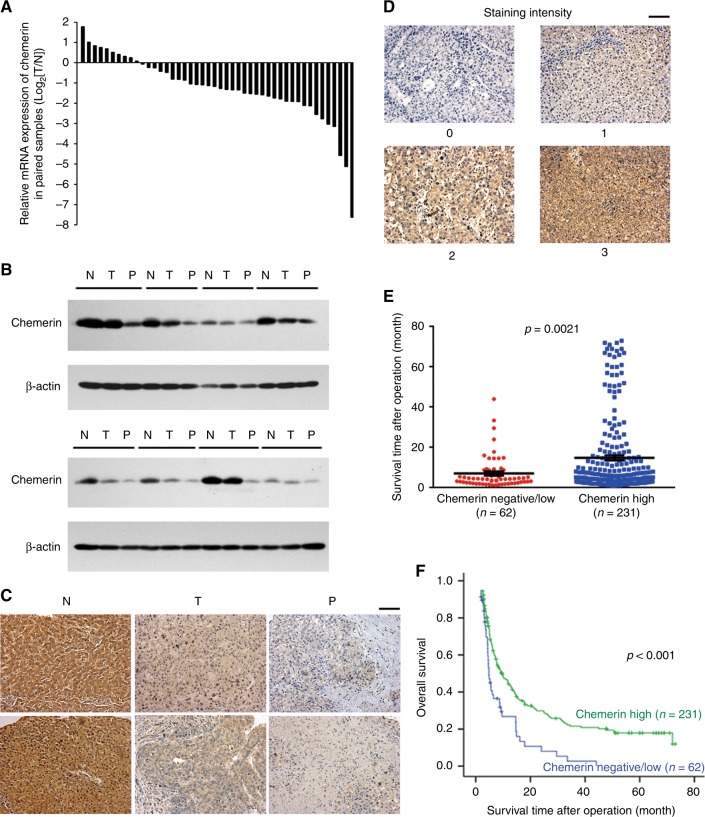
Expression pattern and clinical significance of chemerin in HCC. **a** Expression of chemerin mRNA in 46 pairs of HCC tissues and adjacent non-tumourous liver tissues. **b** Expression of chemerin proteins in primary HCC tissues (T), portal vein tumour thrombi (P) and paired normal liver tissues (N). **c** Representative immunohistochemical staining for chemerin in paired normal liver (N), primary HCC (T) and PVTT (P) tissues. Scale bar = 100 µm. **d** Representative immunohistochemical staining for chemerin with different scores (0, 1, 2, 3) in TMA1. Scale bar = 100 µm. **e** Scatter diagram reflecting survival of HCC patients and chemerin expression in TMA1. **f** Kaplan–Meier survival curve of overall survival according to chemerin expression in TMA1, *p* < 0.001

The expression of chemerin mRNA was significantly reduced in 28 of 46 (61%) HCC samples compared to their normal counterparts (Fig. 1a), and the decreased expression of chemerin was further confirmed at the protein level via western blot (Fig. 1b) and immunohistochemistry (Fig. 1c, Supplementary Table S1). To further evaluate the clinical significance of chemerin in HCC, the expression of chemerin was examined by immunohistochemistry in TMA1. Grade 0 showed no positive signal, while Grade 1, 2, 3 showed weak, modest and strong staining for chemerin, respectively (Fig. 1d). On the basis of chemerin expression, the HCC patients were classified into two groups: chemerin negative/low (*n* = 62) and chemerin high (*n* = 231). Figure 1e displayed a significant difference in the mean overall survival between the two groups: 8.8 months for chemerin negative/low group while 15.3 months for chemerin high group (*p* = 0.0021). Kaplan–Meier survival analysis revealed that chemerin high group had better overall survival than chemerin negative/low group (*p* < 0.001, Fig. 1f). The median survival time for chemerin negative/low group was 4.8 months (95%CI: 3.8–5.8), while it was 9.6 months (95%CI: 6.8–12.4) for chemerin high group. Univariate Cox Regression Analysis revealed that chemerin expression significantly influenced the overall survival (*p* < 0.001, 95%CI = 1.393–2.737) of HCC patients. Multivariate Cox Regression Analysis disclosed that chemerin could act as an independent risk factor for overall survival (*p* = 0.001, 95%CI: 1.294–2.558) and disease-free survival (*p* = 0.019, 95%CI = 1.059–1.891) (Supplementary Table S2). Furthermore, chemerin expression was significantly associated with HBV (*p* = 0.035), hepatocirrhosis (*p* = 0.048), and macrophage infiltration (*p* = 0.029) (Supplementary Tables S3 and S4).

Portal vein tumour thrombus (PVTT) is an important poor prognostic factor for HCC, and a strong statistical correlation exists between intrahepatic metastasis and PVTT.^12^ Interestingly, expression of chemerin was negative/low in 16 of 28 (57%) PVTT tissues, which was significantly downregulated compared to the corresponding primary HCC tissues (*p* = 0.014) (Fig. 1b, c), indicating a potential association between low chemerin level and PVTT development.

### Chemerin decreases HCC cell migration and invasion in vitro

The clinical significance of chemerin stimulated us to explore its role in HCC. We found that chemerin receptor CMKLR1 was expressed in both HCC cells and normal liver cells, while chemerin was detected only in HepG2 (Supplementary Figure S1B), a HCC cell line with poor tumourigenic potential.

**Fig. 2:**
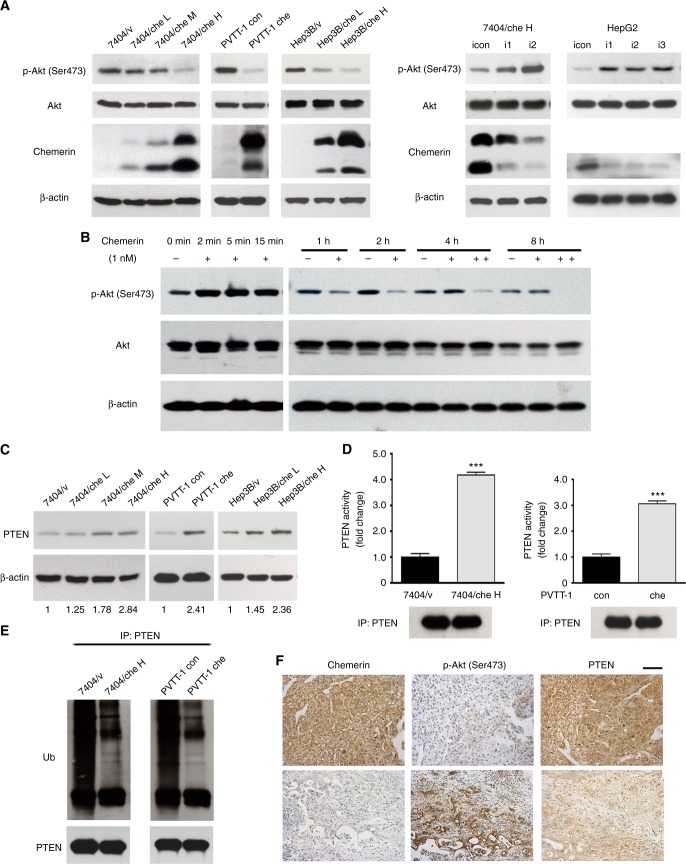
Chemerin decreases migratory and invasive capabilities of HCC cells. Migratory and invasive capabilities of chemerin-overexpressing 7404 cells (**a**) and chemerin knockdown 7404/che H cells (**b**) are examined by Boyden Chamber Assay (upper) and Transwell Invasion Assay (lower), respectively. Quantitation is provided graphically (right), and each column represents the mean (±s.e.m.) number of cells from six randomly selected fields. *p = 0.0142 (migration) and **p = 0.0060 (invasion) for 7404/che L vs. 7404/v, ***p < 0.001 for 7404/che M, H Vs 7404/v, and 7404/che H i1, i2 vs. 7404/che H icon, Unpaired *t* test. **c** Effect of antibody (Ab) neutralisation on migration (1 μg/ml and 10 μg/ml Ab) and invasion (5 μg/ml and 50 μg/ml Ab) of 7404/che H cells. For migration, **p = 0.001 for 1 μg/ml Ab vs. IgG con, ***p < 0.001 for 10 μg/ml Ab vs. IgG con, Unpaired *t* test with Welch correction. For invasion, **p = 0.0048 for 5 μg/ml Ab vs. IgG con, ***p < 0.001 for 50 μg/ml Ab vs. IgG con, Unpaired *t* test. Scale bar = 100 µm

**Fig. 3:**
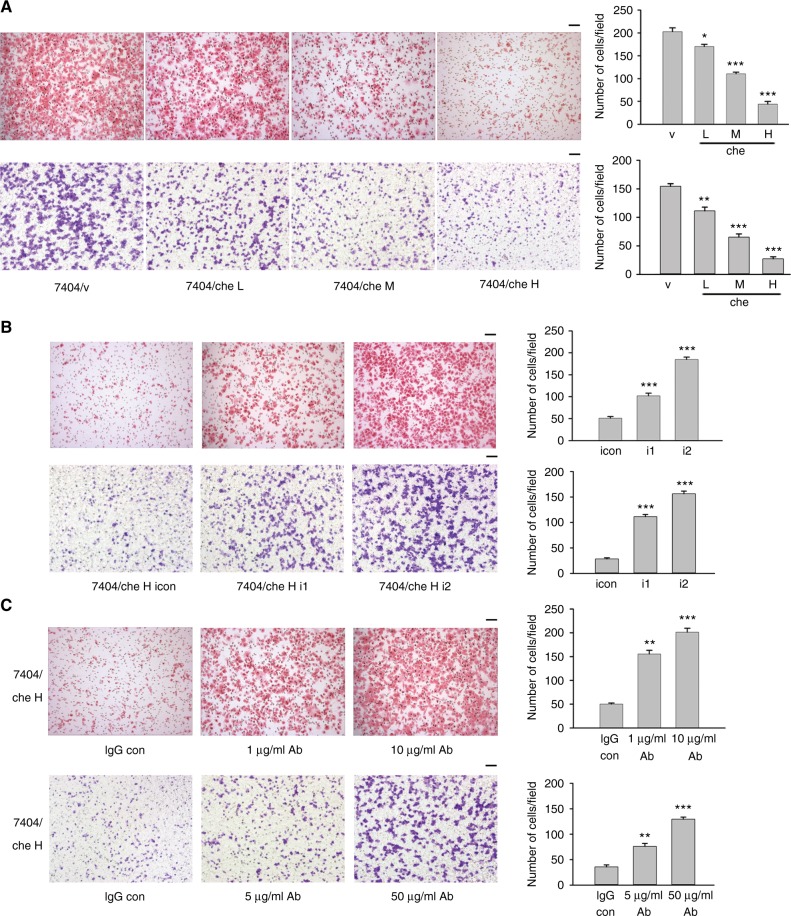
Regulation of PTEN-Akt axis by chemerin. **a** Expression of p-Akt (Ser473), total Akt, chemerin and β-actin in chemerin-overexpressing cells, chemerin knockdown cells and their respective control cells. **b** 7404 cells are treated with 1 nM chemerin, and cells are collected at the indicated time points. “−”, control, “+”, chemerin added, “++”, fresh media containing 1 nM recombinant chemerin was replenished every 2 h. **c** Expression of PTEN in control and chemerin-overexpressing HCC cells. Relative quantification of PTEN expression is listed below. **d** PTEN activity of control and chemerin-overexpressing HCC cells. ***p < 0.001 for 7404/che H vs. 7404/v, PVTT-1 che vs. PVTT-1 con, Unpaired *t* test. Precipitated PTEN is shown below. **e** PTEN ubiquitination in control and chemerin-overexpressing HCC cells. **f** Representative immunohistochemical staining for chemerin, p-Akt (Ser473) and PTEN of the same tissue cores (TMA1). Scale bar = 100 µm

We overexpressed chemerin in 7404, PVTT-1 and Hep3B cells (Fig. 3a, Supplementary Figure S1C). Chemerin significantly suppressed migration and invasion of HCC cells (Fig. 2a, Supplementary Figures S2A and S2B), but showed no effect on cell proliferation (Supplementary Figure S1D) or apoptosis (Supplementary Figure S1E and S1F). In contrast, knockdown of chemerin in 7404/che H and HepG2 cells increased cell migration and invasion (Fig. 2b, Supplementary Figure S2C). Neutralising antibody effectively increased the migratory and invasive capability of 7404/che H cells in a dose-dependent manner (Fig. 2c). Moreover, recombinant chemerin markedly and dose-dependently reduced the migration ability of 7404 and PVTT-1 cells (Supplementary Figure S2D). These data suggested that the inhibition of HCC cell migration and invasion was attributed to secreted chemerin.

### Chemerin inhibits HCC cell migration and invasion through negative regulation of p-Akt (Ser473) by PTEN

To investigate the underlying mechanisms of chemerin, its known downstream signaling was examined. The level of p-Akt (Ser473) was downregulated in chemerin-overexpressing 7404, PVTT-1 and Hep3B cells, while it was increased in chemerin knockdown 7404/che H and HepG2 cells (Fig. 3a), which was consistent with the altered migratory and invasive capabilities of these cells. However, p-Akt (Thr308) showed little alteration (Supplementary Figure S3C). Akt was previously reported to be activated by chemerin, a typical signaling transduction downstream of GPCR.^13,14^ Consistently, we observed Akt activation in short-term exposure to chemerin (Fig. 3b). However, in the long-term treatment, the level of p-Akt (Ser473) declined by 1 h, hitting a nadir at 2 h, then reverted to the resting level at 4 h. However, when the medium was changed every 2 h and replenished with 1 nM chemerin, the decreased level of p-Akt (Ser473) was noted even at 8 h (Fig. 3b). Therefore, our experiments suggested that prolonged exposure to chemerin inhibited Akt, while short-term exposure to chemerin activated Akt. Furthermore, constitutively active (CA)-Akt successfully rescued the impaired migration and invasion capability of 7404/che H cells (Supplementary Figure S3A).

We also found that active extracellular MMP1, a molecule downstream of Akt, was significantly decreased in chemerin-overexpressing HCC cells, while increased in chemerin knockdown HCC cells (Supplementary Figure S3B), which may be responsible for decreased migration and invasion and of HCC cells affected by chemerin via Akt. Taken together, these data suggested that chemerin inhibited HCC cell migration and invasion through negative regulation of Akt.

To further clarify the negative regulation of Akt by chemerin, we examined the status of PTEN, one of the major regulators of Akt. We found that the expression of PTEN was moderately elevated in chemerin-overexpressing cells (Fig. 3c), while it was downregulated when chemerin was silenced (Supplementary Figure S4B). However, activity of PTEN changed significantly but inversely in chemerin-overexpressing and knockdown HCC cells (Fig. 3d and Supplementary Figure S3D). Levels of p-PTEN (Ser380/Thr382/383), the crucial phosphorylation sites influencing PTEN stability and activity were not affected by chemerin (Supplementary Figure S3C). In contrast, ubiquitination of PTEN, another important modification modulating PTEN stability^15^ and activity,^16,17^ was significantly decreased in chemerin-overexpressing HCC cells (Fig. 3e), and increased upon chemerin knockdown (Supplementary Figure S3E). Cycloheximide treatment revealed that the half-life of endogenous PTEN was prolonged by chemerin overexpression (Supplementary Figure S3F), which was consistent with reduced ubiquitination of PTEN in chemerin-overexpressing HCC cells.

To identify the relationship between PTEN, Akt and chemerin, we examined the level of p-Akt (Ser473) and PTEN in HCC TMA1 by immunohistochemistry, and analyse their correlation with chemerin expression. We found that high expression of chemerin was significantly associated with low level of p-Akt (Ser473) and high expression of PTEN, which was consistent with our observation in HCC cells (Fig. 3f, Supplementary Table S5).

### CMKLR1-PTEN axis is responsible for downregulation of p-Akt (Ser473)

**Fig. 4:**
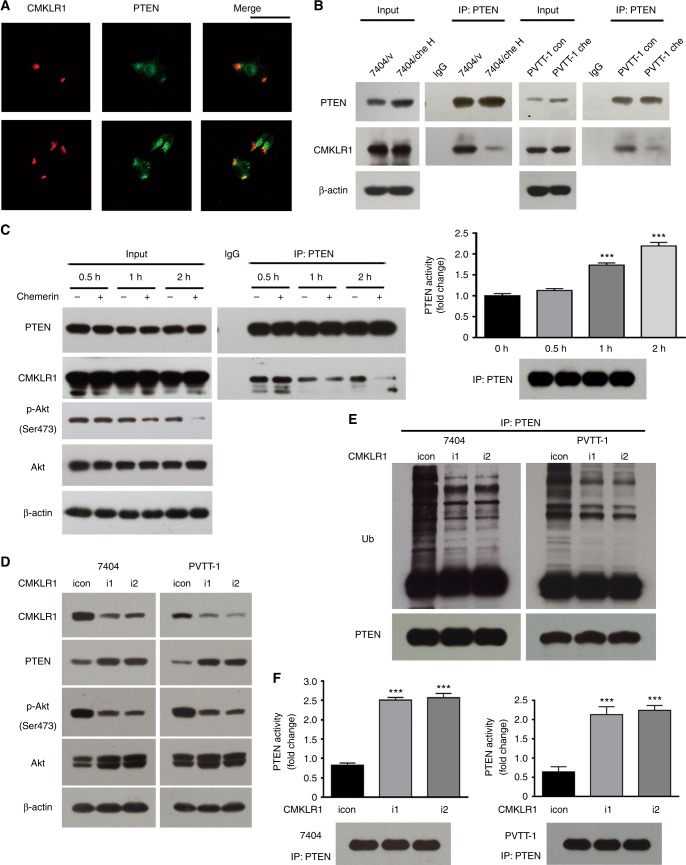
CMKLR1-PTEN axis is responsible for downregulation of p-Akt (Ser473). **a** Immunofluoresent staining of PTEN and CMKLR1 in HCC cells. Scale bar = 100 µm. **b** PTEN–CMKLR1 interaction in chemerin-overexpressing and control cells is examined by immunoprecipitation and western blot. **c** Left, 7404 cell lysates of either control or chemerin-treated cells are immunoprecipitated with control IgG or anti-PTEN antibody and then subjected to western blot analysis. Right, PTEN activity in chemerin-treated cells. ***p < 0.001 for 1 h vs. 0 h, 2 h vs. 0 h, Unpaired *t* test. Precipitated PTEN is shown below. **d** Expression of CMKLR1, PTEN, p-Akt (Ser473), Akt and β-actin in control cells and CMKLR1 knockdown cells. **e** Ubiquitination of PTEN in control and CMKLR1 knockdown cells. **f** PTEN activity in control and CMKLR1 knockdown cells. ***p < 0.001 for i1/i2 vs. icon, Unpaired *t* test. Precipitated PTEN is shown below

Since CMKLR1 mediated intracellular signal transduction induced by chemerin, the interaction between PTEN and CMKLR1 was examined. PTEN and CMKLR1 demonstrated partial co-localisation in HCC cell (Fig. 4a). Their interaction was also detected by GST-pulldown (Supplementary Figure S4A), and the endogenous interaction between CMKLR1 and PTEN was further confirmed by immunoprecipitation, while this interaction was remarkably weakened in chemerin-overexpressing cells compared to control cells (Fig. 4b). In contrast, chemerin knockdown strengthened PTEN–CMKLR1 interaction (Supplementary Figure S4B), suggesting that PTEN dissociated from CMKLR1 in the presence of chemerin. To further clarify the effect of chemerin on PTEN–CMKLR1 interaction, long-term chemerin treatment was performed to mimic the situation in chemerin-overexpressing cells. PTEN–CMKLR1 interaction markedly decreased by 2 h (Fig. 4c), which paralleled the decreased level of p-Akt (Ser473). However, the expression of PTEN was not changed during chemerin treatment, which may be attributed to the low dose of extraneous recombinant chemerin (1 nM). Although PTEN ubiquitination was not detected due to the potential disturbing effect of MG132, PTEN activity gradually increased after 30 min, and reached a peak at 2 h (Fig. 4c), which was coincident with the lowest level of p-Akt (Ser473) and the weakest PTEN–CMLKR1 interaction.

To ulteriorly confirm the involvement of CMKLR1-PTEN axis in chemerin function, PTEN expression was knocked down by RNAi. PTEN knockdown significantly upregulated the level of p-Akt (Ser473), and increased the migration and invasion capability of chemerin-overexpressing HCC cells (Supplementary Figure S4C and S4D). We also modulate the expression of CMKLR1 in HCC cells, and found that CMKLR1 knockdown resulted in elevated expression of PTEN in the absence of chemerin (Fig. 4d), associated with weakened ubiquitination of PTEN, elevated PTEN activity, decreased p-Akt (Ser473) level, and suppressed migration and invasion of HCC cells, indicating that PTEN released from CMKLR1 is less ubiquitinated and more active (Fig. 4d–f, Supplementary Figure S4E). These data suggested that chemerin affected PTEN expression and activity through its cognate receptor CMKLR1.

### Overexpression of chemerin decreases both distant and intrahepatic metastasis of HCC cells *in vivo*

**Fig. 5:**
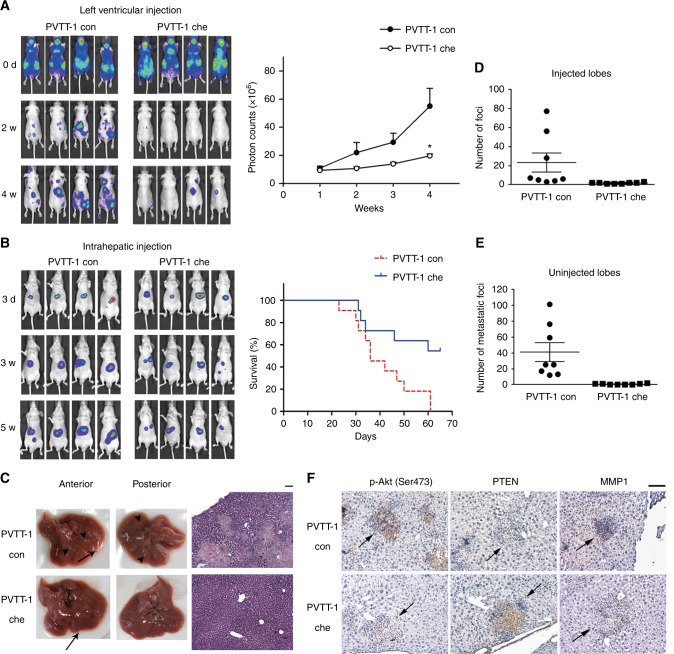
Overexpression of chemerin decreases metastases of HCC cells. **a** Left, metastatic capability of luciferase-labelled PVTT-1 con and chemerin-overexpressing PVTT-1 che cells is examined using left ventricular injection model. Images obtained at indicated time points are shown. Right, mean value of photon counts in PVTT-1 con group and PVTT-1 che group. The data are expressed as mean (±s.e.m.), and are representative of at least two independent experiments, *n* = 8, *p = 0.0272 for PVTT-1 che vs. PVTT-1 con group at the 4th week, Unpaired *t* test with Welch correction. **b** Left, representative images of the mice intrahepatically injected with luciferase-labelled PVTT-1 con or PVTT-1 che cells. Right, survival curves for the mice in PVTT-1 con and PVTT-1 che group. **c** Representative photos of livers (left) and H&E stained liver tissue sections (right) of mice from PVTT-1 con and PVTT-1 che group receiving intrahepatic injection. Foci at injection sites are indicated by arrow, and metastatic foci are indicated by arrowhead. Scale bar = 100 µm. Number of superficial tumour foci on the injected (**d**) and uninjected lobes (**e**) of PVTT-1 con and PVTT-1 che group. The data are expressed as mean (±s.e.m.), *n* = 8 mice per group, *p* < 0.001 for both injected lobes and uninjected lobes, Mann–Whitney test. **f** Representative immunohistochemical staining for p-Akt (Ser473) and PTEN of the same foci (indicated by arrows), and MMP1 of different foci on sequential liver tissue sections of the mice in PVTT-1 con and PVTT-1 che group

Considering the close association between migration, invasion and metastasis, the effect of chemerin on HCC metastasis was examined using luciferase-based murine model. In left ventricular injection model, luciferase-labelled PVTT-1 con and PVTT-1 che cells were injected into the left ventricles of nude mice, respectively. Significant metastatic foci appeared in the mice injected with PVTT-1 con cells two weeks after injection, while rare visible foci could be detected in PVTT-1 che group (Fig. 5a). The distant metastases in PVTT-1 con group were much more wide-spread compared to the PVTT-1 che group 4 weeks after injection, as assessed by mean fluorescence intensity (Fig. 5a).

We also employed intrahepatic injection model, which more closely mimicked the physiological progression of HCC metastasis. Luciferase-labelled PVTT-1 con or PVTT-1 che cells were injected into the left hepatic lobes of nude mice, respectively, and subsequent weekly examination was performed to measure the fluorescent signals. The signal markedly increased in PVTT-1 con group, while it increased more slowly or even was weakened in PVTT-1 che group (Fig. 5b). Consistently, the mice in PVTT-1 che group had a longer survival than those in PVTT-1 con group. Mean survival time for PVTT-1 con and PVTT-1 che group was 41 days (95%CI: 34–48) and 54 days (95%CI: 45–62), respectively (*n* = 11 mice per group, *p* = 0.0125, Fig. 5b). Intrahepatic metastases were assessed by counting the superficial foci of the uninjected liver lobes and histopathological analysis of H&E stained liver sections (Fig. 5c, e). PVTT-1 con group developed dramatically more tumour foci in both injected and uninjected lobes compared to PVTT-1 che group (Fig. 5d, e), which was consistent with the stronger fluorescent signals in PVTT-1 con group (Fig. 5b). Furthermore, we found that the level of p-Akt (Ser473) and MMP1 of foci was high, while the expression of PTEN was low in PVTT-1 con group. In contrast, the foci in PVTT-1 che group demonstrated reverse expression pattern, which was consistent with HCC cells in vitro (Fig. 5f). Therefore, the two different murine models revealed that overexpression of chemerin in HCC cells led to effective suppression of intrahepatic and extrahepatic metastases, associated with low p-Akt (Ser473), MMP1 and high PTEN expression.

### Investigation of the therapeutic potential of chemerin in HCC

**Fig. 6:**
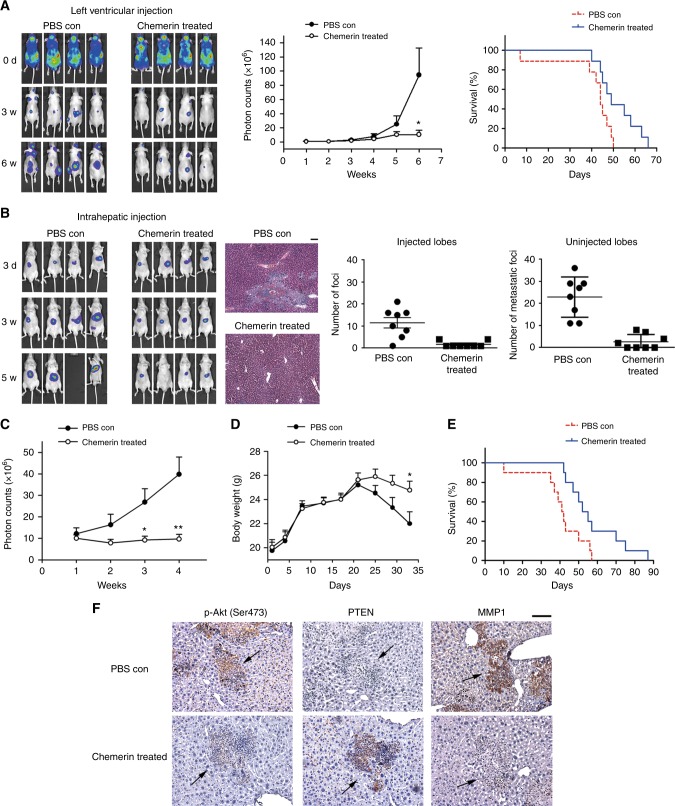
Chemerin treatment effectively inhibits HCC metastasis and improves the survival of HCC-bearing mice. **a** Effect of chemerin is examined in left ventricular injection model. Left, representative images. Middle, mean value of photon counts, data are expressed as mean (±s.e.m.) and are representative of at least two independent experiments. *n* = 9 mice per group, *p = 0.0379 at the 6th week, Mann–Whitney test. Right, survival curves of the mice. **b** Effect of chemerin is examined in mice receiving intrahepatic injection. Left, representative images. Middle, H&E stained liver tissue sections. Right, superficial foci on injected lobes (left) and uninjected lobes (right) of PBS con or chemerin treated group were counted. The data are expressed as mean (±s.e.m.) and representative of at least two independent experiments. *n* = 8 mice per group, *p* = 0.0012 for the injected lobes, and *p* < 0.001 for the uninjected lobes, Unpaired *t* test. **c** Luciferase signals [mean photon counts (±s.e.m.)] in the intrahepatic injection model. The data are representative of at least two independent experiments, *n* = 10 mice per group, *p = 0.0213 at the 3rd week, and **p = 0.0047 at the 4th week, Unpaired *t* test with Welch correction. **d** Weight [mean (±s.e.m.)] of mice, *n* = 10 mice per group, *p = 0.0348 for chemerin treated group vs. PBS con group at 33rd day, Unpaired *t* test. **e** Survival curves of the mice undergoing chemerin treatment in intrahepatic injection model. **f** Representative immunohistochemical staining for p-Akt (Ser473) and PTEN of the same foci (indicated by arrows), and MMP1 of different foci on sequential liver tissue sections of the mice in PBS con and chemerin treated group in intrahepatic injection model. Scale bar = 100 µm

The above studies suggested a potential application of chemerin in HCC therapy, thus we purified recombinant chemerin as previously described with little modification^8^(SupplementaryMaterials,Methods) (Supplementary Figure S5A) and evaluated its therapeutic potential using different murine models. In left ventricular injection model, intraperitoneal injection every other day of recombinant chemerin inhibited the development of distant metastases and effectively prolonged survival of the mice (Fig. 6a). Median survival time for PBS con group was 44 days (95%CI: 41–47), while it was 49 days (95%CI: 43–55) for chemerin-treated group (*n* = 9 mice per group, *p* = 0.0316). In intrahepatic injection model, the mice were intrahepatically injected with PVTT-1 luci cells, and recombinant chemerin treatment (0.5 mg/kg) was begun 3 days after intraperitoneal injection and given every other day. Similarly, chemerin treatment significantly reduced both the incidence and spreading of intrahepatic metastasis, as assessed by counting superficial foci on the uninjected liver lobes, fluorescence intensity and H&E staining of the liver sections (Fig. 6b, c). Furthermore, chemerin treatment markedly hindered weight loss of tumour-bearing mice during HCC progression (Fig. 6d), and extended survival of the mice (Fig. 6e). Median survival for PBS con and chemerin treated group was 41 days (95%CI: 36–46) and 52 days (95%CI: 44–60), respectively (*n* = 10 mice per group, *p* = 0.0186).

We also examined expression of p-Akt (Ser473), PTEN and MMP1 in the intrahepatic injection model. The foci in chemerin-treated group demonstrated higher PTEN level, while lower expression of p-Akt (Ser473) and MMP1 compared to the foci in PBS con group (Fig. 6f). Therefore, positive regulation of PTEN, and subsequent suppression of Akt and MMP1 possibly contributed to suppression of HCC metastasis by chemerin in vivo.

In the previous report,^18^ chemerin expression was significantly correlated with the infiltration of dendritic cells (DC) and natural killer (NK) cells in HCC. Considering the association between chemerin expression and macrophage infiltration (Supplementary Table S4), we detected the effect of chemerin treatment on macrophage and neutrophil recruitment. As shown in Supplementary Figure S5B, infiltration of Myeloperoxidase (MPO)-positive neutrophils and CD68-positive monocytes was increased in the livers of chemerin-treated mice, which was further identified by the elevated expression of marker genes (Supplementary Figure S5C). These data demonstrated that chemerin could significantly extend the survival and hinder the weight loss of the nude mice inoculated with HCC cells, through inhibition of HCC metastasis and recruitment of immune cells.

## Discussion

Chemerin was initially discovered as a retinoid responsive gene present in psoriatic skin lesions in 1997.^4^ Subsequent studies identified chemerin as a ligand for the orphan receptor CMKLR1,^5^ which is a seven-pass transmembrane GPCR related to the chemoattractant C3a and C5a complement receptors and the bacterial peptide fMLP receptor.^19^ CMKLR1 expression appears to be restricted to dendritic cells (DCs), neutrophils and macrophages. Accordingly, the function of chemerin/CMKLR1 was firstly investigated in immune system, establishing the chemoattractant activity of chemerin in immune response.^5^ However, the role of chemerin in cancer is largely unknown. Decreased expression of chemerin has been reported in a few cancers, including uterine fibroids,^20^ skin squamous cell carcinoma,^21^ liver cancer,^18,22^ and melanoma.^8^ The study in melanoma suggested that downregulation of chemerin may be an important mechanism of tumour immune evasion. A recent study identified chemerin as a negative regulator of HCC-associated inflammation and immunosuppression.^22^ Consistently, we reported that expression of chemerin in HCC was decreased compared to cancer-adjacent normal tissues, and chemerin treatment enhanced immune cell recruitment to the liver, restraining HCC progression. Furthermore, we demonstrated that chemerin significantly reduced migration, invasion and metastasis of HCC cells, which revealed the immune-independent function of chemerin in cancer.

Previous study reported that ligation of chemerin to CMKLR1 activated Akt,^13^ which appeared inconsistent with our observation. However, the previous studies showed that chemerin induced Akt activation within a relative short time period in human endothelial cells^14^ and chondrocytes^13^ (<30 min). We also observed that short-term exposure to chemerin activated Akt (<30 min), followed by a decline of p-Akt (Ser473) below basal level (>30 min) (Fig. 3b). Biphasic regulation of receptor-associated signaling has been reported previously, including IGF-1 initially increasing and subsequently decreasing the phosphorylation of Erk1/2 in skeletal muscle cells, associated with cell differentiation.^23^ Another study revealed a time-dependent biphasic cAMP response after stimulation of LGR7 by human gene 2 (H2) relaxin, associated with signal switching from Gs to Gi.^24^ The biphasic effect of chemerin on Akt phosphorylation may be caused by the alterations in the receptor and receptor-associated signaling.

In this study, we found that PTEN could interact with chemerin receptor CMKLR1, and PTEN–CMKLR1 interaction was weakened by chemerin binding. An explanation for the biphasic regulation of Akt by chemerin is that chemerin initially induces activation of Akt through the canonical GPCR-mediated PI3K pathway; meanwhile, PTEN is released from chemerin-bound CMKLR1, with reduced ubiquitination and promoted activity. PTEN accumulates and ultimately inhibits chemerin-induced PI3K-Akt signaling. Our study also suggests a potential regulation of PTEN in HCC development and chemerin treatment. In normal liver tissues, chemerin helps maintain the regular expression and phosphatase activity of PTEN. When chemerin is downregulated in the development of HCC, PTEN is restricted by CMKLR1, which leads to ubiquitination and suppression of PTEN. Extraneous chemerin activates CMKLR1 and releases PTEN, then the recovered PTEN regains tumour suppressor activity and inhibits Akt, resulting in suppressed migration, invasion and metastasis of HCC cells, at least partially through downregulation of MMP1.^25–27^ This hypothesis is supported by our observation in clinical samples, HCC cells, and animal models of HCC metastasis. However, further studies are required to explore the details of CMKLR1–PTEN interaction, clarify their mutual regulation, and find out more effectors downstream of CMKLR1/PTEN/Akt signaling axis.

Chemokine-based immune therapy has become an attractive therapeutic strategy in the management of HCC. One study has shown that administration of ECI301, an active variant of the CC chemokine ligand 3, augmented the antitumour effect of radiofrequency ablation in a CCR1-dependent manner.^28^ In another study, administration of interleukin-12 was shown to enhance the therapeutic efficacy of dendritic cell-based tumour vaccines in moues hepatocellular carcinoma.^29^ In our study, administration of chemerin in HCC-bearing nude mice significantly restrained the development of both distant and intrahepatic metastasis. These antitumour effects were not only attributed to recruitment of immune cells, but also due to inhibition of metastatic capability of HCC cells. In addition, because chemerin is naturally produced by normal hepatocytes, appropriate administration of chemerin is nontoxic to normal tissues, which is optimal for drug development.

In conclusion, our study has revealed a novel suppressive role of chemerin in HCC, through its inhibition of HCC metastasis and its chemoattractive capability to recruit immune cells. We also revealed a negative regulation of Akt by chemerin-CMKLR1-PTEN axis, which expand our understanding of chemerin-mediated signaling. The dual effects of chemerin on both HCC cells and tumour microenvironment make it a promising candidate for HCC therapy.

## Electronic supplementary material

Supplementary Tables

Supplementary Materials and Methods

Supplementary Figure 1

Supplementary Figure 2

Supplementary Figure 3

Supplementary Figure 4

Supplementary Figure 5

## Data Availability

All the data generated or analysed during this study are included in this published article [and its supplementary information files].

## References

[1] Ferlay J (2015). Cancer incidence and mortality worldwide: sources, methods and major patterns in GLOBOCAN 2012. Int. J. Cancer.

[2] Ono T, Yamanoi A, Nazmy El Assal O, Kohno H, Nagasue N (2001). Adjuvant chemotherapy after resection of hepatocellular carcinoma causes deterioration of long-term prognosis in cirrhotic patients: metaanalysis of three randomized controlled trials. Cancer.

[3] Yao FY (2001). Liver transplantation for hepatocellular carcinoma: expansion of the tumor size limits does not adversely impact survival. Hepatology.

[4] Nagpal S (1997). Tazarotene-induced gene 2 (TIG2), a novel retinoid-responsive gene in skin. J. Invest. Dermatol..

[5] Wittamer V (2003). Specific recruitment of antigen-presenting cells by chemerin, a novel processed ligand from human inflammatory fluids. J. Exp. Med..

[6] Zabel BA (2006). Chemokine-like receptor 1 expression by macrophages in vivo: regulation by TGF-beta and TLR ligands. Exp. Hematol..

[7] Parolini S (2007). The role of chemerin in the colocalization of NK and dendritic cell subsets into inflamed tissues. Blood.

[8] Pachynski RK (2012). The chemoattractant chemerin suppresses melanoma by recruiting natural killer cell antitumor defenses. J. Exp. Med..

[9] Tada H (2001). Systemic IFN-beta gene therapy results in long-term survival in mice with established colorectal liver metastases. J. Clin. Invest..

[10] Li Y (2001). Establishment of cell clones with different metastatic potential from the metastatic hepatocellular carcinoma cell line MHCC97. World J. Gastroenterol..

[11] Li Y (2003). Establishment of a hepatocellular carcinoma cell line with unique metastatic characteristics through in vivo selection and screening for metastasis-related genes through cDNA microarray. J. Cancer Res. Clin. Oncol..

[12] Toyosaka A (1996). Pathologic and radiographic studies of intrahepatic metastasis in hepatocellular carcinoma; the role of efferent vessels. Hpb. Surg..

[13] Berg V (2010). Human articular chondrocytes express ChemR23 and chemerin; ChemR23 promotes inflammatory signalling upon binding the ligand chemerin(21-157). Arthritis Res. Ther..

[14] Kaur J, Adya R, Tan BK, Chen J, Randeva HS (2010). Identification of chemerin receptor (ChemR23) in human endothelial cells: chemerin-induced endothelial angiogenesis. Biochem. Biophys. Res. Commun..

[15] Yim EK (2009). Rak functions as a tumor suppressor by regulating PTEN protein stability and function. Cancer Cell..

[16] Maccario H, Perera NM, Gray A, Downes CP, Leslie NR (2010). Ubiquitination of PTEN (phosphatase and tensin homolog) inhibits phosphatase activity and is enhanced by membrane targeting and hyperosmotic stress. J. Biol. Chem..

[17] Lee JT (2013). RFP-mediated ubiquitination of PTEN modulates its effect on AKT activation. Cell Res..

[18] Lin W, Chen YL, Jiang L, Chen JK (2011). Reduced expression of chemerin is associated with a poor prognosis and a lowed infiltration of both dendritic cells and natural killer cells in human hepatocellular carcinoma. Clin. Lab..

[19] Samson M (1998). ChemR23, a putative chemoattractant receptor, is expressed in monocyte-derived dendritic cells and macrophages and is a coreceptor for SIV and some primary HIV-1 strains. Eur. J. Immunol..

[20] Zaitseva M, Vollenhoven BJ, Rogers PA (2008). Retinoids regulate genes involved in retinoic acid synthesis and transport in human myometrial and fibroid smooth muscle cells. Hum. Reprod..

[21] Zheng Y (2008). Downregulation of tazarotene induced gene-2 (TIG2) in skin squamous cell carcinoma. Eur. J. Dermatol..

[22] Lin Y (2017). Chemerin has a protective role in hepatocellular carcinoma by inhibiting the expression of IL-6 and GM-CSF and MDSC accumulation. Oncogene.

[23] Adi S, Bin-Abbas B, Wu NY, Rosenthal SM (2002). Early stimulation and late inhibition of extracellular signal-regulated kinase 1/2 phosphorylation by IGF-I: a potential mechanism mediating the switch in IGF-I action on skeletal muscle cell differentiation. Endocrinology.

[24] Halls ML, Bathgate RA, Summers RJ (2005). Signal switching after stimulation of LGR7 receptors by human relaxin 2. Ann. N. Y. Acad. Sci..

[25] Xu X, Qin J, Liu W (2014). Curcumin inhibits the invasion of thyroid cancer cells via down-regulation of PI3K/Akt signaling pathway. Gene.

[26] Tahara H (2013). Transforming growth factor-α activates pancreatic stellate cells and may be involved in matrix metalloproteinase-1 upregulation. Lab Invest..

[27] Lin CY (2014). A dual tyrosine kinase inhibitor lapatinib suppresses overexpression of matrix metallopeptidase 1 (MMP1) in endometrial cancer. J. Mol. Med..

[28] Iida N (2010). Antitumor effect after radiofrequency ablation of murine hepatoma is augmented by an active variant of CC Chemokine ligand 3/macrophage inflammatory protein-1alpha. Cancer Res..

[29] Tatsumi T (2001). Administration of interleukin-12 enhances the therapeutic efficacy of dendritic cell-based tumor vaccines in mouse hepatocellular carcinoma. Cancer Res..

