# Supplementary material for: Chemerin suppresses hepatocellular carcinoma metastasis through CMKLR1-PTEN-Akt axis
Source: Br J Cancer. 2018 May 2;118(10):1337–48. doi: 10.1038/s41416-018-0077-y (PMC5959946; doi:10.1038/s41416-018-0077-y)
Supplement: Supplementary file 1 — Supplementary Tables [file 41416_2018_77_MOESM1_ESM.docx]

**Supplementary Tables**

**Supplementary Table S1 Comparison of chemerin expression in normal liver and paired HCC tissues**

| N-T (score) | n (cases) | *P* value |
| --- | --- | --- |
| 3 | 4 | <0.001^b^ |
| 2 | 37 |  |
| 1 | 93 |  |
| 0 | 52 |  |
| -1 | 7 |  |
| -2 | 1 |  |

TMA2 were immunostained with anti-human chemerin antibody, and two experienced pathologists evaluated the immunoreactivity and histological appearance of all tissue samples in the microarray.The intensity of chemerin staining was scored on a scale of 0 to 3, with 0 being no staining, 1 as weak intensity, 2 as moderate intensity and 3 as strongest intensity. The value of “N-T” equals to the score of normal liver tissue minus that of the paired HCC tissue. One sample t-test.

^b^ p<0.001

**Supplementary Table S2 Univariate and Multivariate Analysis of Factors Associated with Survival of HCC patients**

|  | **Overall Survival** | | | | **Disease-free Survival** | | | |
| --- | --- | --- | --- | --- | --- | --- | --- | --- |
|  | **Univariate analysis** | | **Multivariate analysis** | | **Univariate analysis** | | **Multivariate analysis** | |
| Factor | P value | HR(95% CI) | P value | HR(95%  CI) | P value | HR(95% CI) | P value | HR(95% CI) |
| Gender: male v female | 0.06 | 0.376- 1.020 | NS |  | 0.041 | 0.431-  0.984 | NS |  |
| Age: <51 v ≥51 years | 0.014^a^ | 1.079- 1.979 | 0.046^a^ | 1.006-  1.868 | 0.053 | 0.997- 1.653 | NS |  |
| Liver Cirrhosis: yes v no | 0.37 | 0.829- 1.655 | NS |  | 0.309 | 0.871- 1.548 | NS |  |
| Tumor Size: >5 v≤5 | 0.004^a^ | 1.367- 4.902 | 0.003^a^ | 1.399- 5.038 | 0.008^a^ | 1.187- 3.304 | NS |  |
| Tumor Number: single v multiple | 0.969 | 0.616- 1.592 | NS |  | 0.758 | 0.630- 1.399 | NS |  |
| TNM: stages I-II v III-IV | 0.002^a^ | 0.293- 0.756 | NS |  | 0.003^a^ | 0.410- 0.876 | 0.006^a^ | 0.411-  0.858 |
| Tumor encapsulation: complete v none | 0.138 | 0.175- 1.273 | NS |  | 0.155 | 0.273- 1.229 | NS |  |
| Chemerin: Low v High | <0.001^b^ | 1.393- 2.737 | 0.001^a^ | 1.294- 2.558 | 0.011^a^ | 1.088-  1.940 | 0.019^a^ | 1.059-  1.891 |

In TMA1, ^a^ p<0.05, ^b^ p<0.001

**Supplementary Table S3 Relationship between chemerin expression**

**and clinical features in hepatocellular carcinoma**

| Clinical characteristic | Chemerin  Negative or Low | expression  Moderate or High | *P* value |
| --- | --- | --- | --- |
| HBV |  |  |  |
| Yes | 49 | 148 | 0.035^a^ |
| No | 13 | 80 |  |
| Tumor character |  |  |  |
| Single nodule | 26 | 106 | 0.545 |
| Multiple nodules | 27 | 104 |  |
| Mixed | 1 | 12 |  |
| Peplos |  |  |  |
| No | 46 | 172 | 1.000 |
| Complete | 2 | 6 |  |
| Incomplete | 14 | 51 |  |
| Cell origin |  |  |  |
| Hepatic cell | 55 | 194 | 0.189 |
| Biliary cell | 4 | 9 |  |
| Mixed | 3 | 28 |  |
| Hepatocirrhosis |  |  |  |
| Yes | 54 | 174 | 0.048^a^ |
| No | 8 | 57 |  |
| TNM |  |  |  |
| II | 6 | 34 | 0.305 |
| IIIa, IIIb, IIIc, IV | 56 | 197 |  |
| Tumor size |  |  |  |
| ≤5 | 4 | 19 | 0.794 |
| >5 | 58 | 212 |  |

^a^ p<0.05

**Supplementary Table S4 Relationship between chemerin expression and macrophages in hepatocellular carcinoma**

|  | Chemerin  Negative or Low | | expression  Moderate or High | *P* value |
| --- | --- | --- | --- | --- |
| Mean value of macrophage | | 240.5054 | 308.0769 | 0.029^a^ |

The number of macrophages in each tissue cores of TMA1 is counted based on CD68 staining. Mean value of macrophage=the number of total macrophges/the number of tissue cores.

^a^ p<0.05

**Supplementary Table S5 Relationship between chemerin expression and p-Akt (Ser473) and PTEN level in hepatocellular carcinoma**

| Gene expression | Chemerin  Negative or Low | expression  Moderate or High | *P* value |
| --- | --- | --- | --- |
| p-Akt (Ser473) |  |  |  |
| Negative or low | 27 | 183 | <0.001^b^ |
| Moderate or high | 35 | 48 |  |
| PTEN |  |  |  |
| Negative or low | 38 | 77 | <0.001^b^ |
| Moderate or high | 24 | 154 |  |

In TMA1, Score 0, negative; 1, low; 2, moderate; 3, high.

^b^ p<0.001
