# Supplementary material for: Chemerin suppresses hepatocellular carcinoma metastasis through CMKLR1-PTEN-Akt axis
Source: Br J Cancer. 2018 May 2;118(10):1337–48. doi: 10.1038/s41416-018-0077-y (PMC5959946; doi:10.1038/s41416-018-0077-y)
Supplement: Supplementary file 2 — Supplementary Materials and Methods [file 41416_2018_77_MOESM2_ESM.docx]

**Supplementary Figure Legends**

**Supplementary Figure S1.** Expression of chemerin in HCC cells, and the effect of chemerin on cell growth and apoptosis. (**A**) Expression of chemerin mRNA in MHCC97 cells (P) and three subclones (L, H, M). (**B**) Expression of CMKLR1 and chemerin in different liver cells and HCC cells. THP-1 and Jurket cells were used as positive control for CMKLR1 expression. (**C**) Extracellular chemerin of chemerin-overexpressing/knockdown cells and their respective control cells is examined by ELISA. ***p<0.001 for both chemerin-overexpressing cells/chemerin knockdown cells Vs control cells, Unpaired t-test. (**D**) Cell growth of control cells (7404/v, PVTT-1 con, 7404/che H icon), chemerin-overexpressing cells (7404/che L, M, H, PVTT-1 che) and chemerin knockdown cells (7404/che H i1, 7404/che H i2) is measured by MTT assay. Apoptosis of chemerin-overexpressing cells (**E**) and chemerin knockdown cells (**F**) is examined by TUNEL staining, and white arrowheads indicate TUNEL-positive cells. Scale bar = 100 µm.

**Supplementary Figure S2.** Effect of chemerin on HCC cell migration and invasion. (**A**) Migratory and invasive capabilities of PVTT-1 con and PVTT-1 che cells are examined by Boyden Chamber Assay (upper) and Transwell Invasion Assay (lower). Quantitation is provided graphically (right), and each column represents the mean (±s.e.m.) numbers of cells from six randomly selected fields. ***p<0.001 for PVTT-1 che Vs PVTT con, Unpaired t-test with Welch correction for migration, and Unpaired t-test for invasion. Migratory abilities of chemerin-overexpressing Hep3B cells (**B**), chemerin knockdown HepG2 cells (**C**) and their respective control cells are examined by Boyden Chamber Assay. Each column represents the mean (±s.e.m.) numbers of cells from six randomly selected fields. ***p<0.001 for Hep3B/che L, H Vs Hep3B/v, and HepG2 i1, i2, i3 Vs icon, Unpaired t-test. (**D**) Effect of recombinant chemerin (1nM and 5nM) on the migratory capability of 7404 cells (upper) or PVTT-1 cells (lower). Bar graphs represent mean (±s.e.m.) number of the cells from six randomly selected fields. **p=0.006 for 1nM chemerin treatment Vs control in 7404 and **p=0.009 in PVTT cells, ***p<0.001 for 5nM chemerin treatment Vs control in either 7404 (Unpaired t-test) or PVTT-1 cells (Unpaired t-test with Welch correction). Scale bar = 100 µm.

**Supplementary Figure S3.** chemerin regulates PTEN-Akt axis. (**A**) Left, 7404/che H cells transfected with either empty vector (Mock) or the plasmid expressing constitutively active Akt (CA-Akt) were harvested 24 hours after transfection and analyzed by Western Blot. Increased level of GSK3beta (Ser9) confirmed that the CA-Akt introduced into the cells is active. Right, cell migration and invasion of 7404/che H (Mock), 7404/che (CA-Akt) and 7404/v is examined by Boyden Chamber Assay (upper) and Transwell Invasion Assay (lower). Scale bar = 100 µm. (**B**) Active MMP1 expression in the same amount of TCA precipitates from tissue culture media from indicated cells are analyzed by Western Blot (left), and MMP1 level is examined by immunofluorescence in indicated cells (right), Scale bar = 100 µm. (**C**) Level of p-Akt (Thr308), total Akt, p-PTEN (Ser380/Thr382/383) and β-actin is examined by Western Blot in chemerin-overexpressing HCC cells and control cells. (**D**) PTEN activity is examined in 7404/che H icon, i1 and i2 cells. *p=0.0107 for i1Vs icon, **p=0.0020 for i2 Vs icon, Unpaired t-test. Precipitated PTEN is shown below. (**E**) Impact of chemerin knockdown on PTEN ubiquitination is determined by immunoprecipitation with anti-PTEN antibody, followed by Western Blot analysis using anti-Ub and anti-PTEN antibodies in 7404/che H icon, i1 and i2 cells. (**F**) PVTT-1 con and PVTT-1 che cells are treated with 100 μg/ml chlorhexidine (CHX), cell lysates are collected at indicated time points and then immunoblotted with antibodies against PTEN and β-actin.

**Supplementary Figure S4.** CMKLR1-PTEN axis is involved in chemerin-regulated migration and invasion of HCC cells. (**A**) HCC cell lysates are incubated with GST alone or GST-PTEN/CMKLR1 and immunoprecipitated with Sepharose beads, then analyzed by Western Blot using anti-CMKLR1/PTEN antibodies. (**B**) PTEN-CMKLR1 interaction is examined in 7404/che H icon, i1 and i2 cells by immunoprecipitation. (**C**) Expression of PTEN, p-Akt (Ser473), Akt and β-actin is examined in 7404/che H and PVTT-1 che control cells and PTEN knockdown cells. Cell migration and invasion is examined in PTEN knockdown (**D**), CMKLR1 knockdown (**E**) and their respective control cells. Scale bar = 100 µm.

**Supplementary Figure S5.** Effect of chemerin treatment on immune cells in intrahepatic injection model. (**A**) Ion-exchange chromatograph purification of recombinant human chemerin (S-Sepharose column, GE). Eluted fractions containing purified chemerin at the indicated OD 280 peak and conductivity are collected (left). 7404 cells are treated with purified chemerin (1nM) for 0.5h, 1h and 2h, lysates are harvested and underwent Western Blot analysis (right), and probed for p-Akt (Ser473), total Akt and β-actin. (**B**) Liver tissue sections of both PBS con group and chemerin treated group in the intrahepatic injection model are immunostained with antibodies for the neutrophil marker, Myeloperoxidase (MPO) and monocyte/macrophage marker CD68, and representative pictures are shown. Scale bar = 100 µm. (**C**) Expression of genes related to immune activation and neutrophil infiltration are examined by Real-Time PCR. **p=0.0088 for chemerin treated group Vs PBS con group for Elane (Unpaired t-test), **p=0.0032 for Lactotransferrin (Unpaired t-test) and **p=0.0034 for Integrin alpha M (Unpaired t-test with Welch correction), respectively.

**Supplementary Materials and Methods**

**Plasmids**

The fragment of human chemerin was cloned into either plasmid pcDNA3.1 (Invitrogen) or viral vector pSin-Puro (Addgene) for overexpression of chemerin.

We used both lentiviral-based RNAi [^1^](#_ENREF_1) and pSuper RNAi system for knockdown of human chemerin. As for the lentiviral-based RNAi system, the sequences targeting human chemerin and CMKLR1 were designed by online software (Ambion), and the RNAi target sequences were listed below:

chei1:5'-GAAACCCGAGTGCAAAGTC-3' (NM_002889.3, 366-384)

chei2:5'-GTTCTGCGGGAGGCTGAGG-3' (NM_002889.3, 484-502)

CMKLR1-i1:5'-GATCAAGGCAAGAACCTCT-3'(NM_001142343.1, 1766-1784)

CMKLR1-i2:5'-GAGTTTGAATATAGACGGT-3'(NM_001142343.1, 3800-3818)

For the pSuper RNAi System, the sequences targeting hchemerin were designed by online software (Oligoengine) and were listed as follows:

TIG2i1:5'-GTGGCCCTGGAGGAATTTC-3' (NM_002889.3, 211-229)

TIG2i2:5'-AAGAAACCCGAGTGCAAAG-3' (NM_002889.3, 364-382)

TIG2i3:5'-GGAATTTAAGCTGCAGCAG-3'(NM_002889.3, 321-339)

We used PLKO.1 for PTEN and CMKLR1 knockdown, and the targeting sequences were lised as follows:

PTENi1:5'-AGGCGCTATGTGTATTATTAT-3'(NM_000314.6, 1545-1565)

PTENi2:5'-CGACTTAGACTTGACCTATAT-3'(NM_000314.6, 1094-1114)

For each system, constructs containing non-specific scrambled sequence were used as negative control.

The HA-CA-Akt as well as the corresponding empty vector was gifts from Dr. Guo Li [^2^](#_ENREF_2).

**Semi-quantitative RT-PCR and Real-time PCR**

Preparation of RNA from either cells or tissues samples, as well as the Real-time PCR was performed according to the methods described by Wang et al previously [^3^](#_ENREF_3). The primers used for amplification of the human chemerin gene were as follows:

F: 5’-GGTCCACTGCCCCATAGA-3’, R: 5’-CTTGGAGAAGGCGAACTGT-3’;

As an internal standard, a fragment of human β-actin was amplified using the following primers: F: 5’-GATCATTGCTCCTCCTGAGC-3’, R: 5’-ACTCCTGCTTGCTGATCCAC-3’.

The primers for examination of mouse gene expression were listed as follows:

Neutrophil Elastase (NM_015779.2)

F: 5’-GCACTGGCCTCAGAGATTGT-3’, R: 5’-CCATGGCCAGACATGGAGTT-3’;

ITGAM (NM_001082960.1)

F: 5’-CCACACTAGCATCAAGGGCA-3’, R: 5’-CCCTGATCACCGTGGAGAAG-3’;

Lactotransferrin (NM_008522.3)

F: 5’-GATGACACCCGGAAACCTGT-3’, R: 5’-CGGTCGCTATGACGTACTCC-3’;

β-actin (NM_007393.3)

F: 5’-GATCATTGCTCCTCCTGAGC-3’, R: 5’-ACTCCTGCTTGCTGATCCAC-3’

The relative mRNA level of target genes to that of β-actin in either clinical samples or cells was calculated and analyzed according to the methods described by Wang et al. [^3^](#_ENREF_3).

**Cells**

MHCC97 and three subclones were purchased from ZhongShan Hospital ^[4-6](#_ENREF_4)^. PVTT-1 (CSQT-2) cells were established by our lab as previously described [^7^](#_ENREF_7). HepG2, Hep3B, 7404 and other cells were purchased from cell banks of SIBS. All the cells were maintained in DMEM supplemented with 10% FBS and antibiotics, 10U/ml penicillin G, and 10mg/ml streptomycin. All cells were incubated at 37^o^C in a humidified atmosphere containing 5% CO2.

Both 7404 cells and Hep3B cells were transfected with pcDNA3.1 empty vector and pcDNA3.1-chemerin, respectively. The positive clones were selected by G418, identified by Western Blot, and termed “che L” (low), “che M” (medium) and “che H” (high) according to the expression level of chemerin. PVTT-1 cells were infected with pSin-puro and pSin-puro-chemerin virus, respectively, and the pools were acquired by puromycin selection. To obtain luciferase-labeled PVTT-1 cells (PVTT-1 luci), PVTT-1 cells were infected with luciferase-expressing virus, and the positive cells were enriched by FACS through the GFP marker contained in the viral expression vector of luciferase. The HepG2 cells were transfected with pSuper RNAi constructs containing either TIG2 icon or TIG2 i1/2/3 sequences, respectively, and the corresponding pools were selected with puromycin. 7404/che H cells were infected with che icon and chei 1/2 lentivirus, respectively, and the corresponding pools were selected with puromycin. The effect of chemerin knockdown by RNAi was identified by Western Blot.

**Chemerin treatment on HCC cells**

HCC cells were plated at 70~80% confluence in 24-well plate with DMEM+10% FBS, and were starved overnight before the treatment. The cells were treated with PBS or 1nM chemerin for the indicated duration, then the cells were harvested and the cell lysates were prepared and analyzed by Western Blot.

**Boyden Chamber Assay**

The migration assay was carried out using the 12-well chemotaxis chamber (Neuro Probe) with a filter (pore size: 8μm). 1.0×10^4^~1.0×10^5^ cells (depending on cell type and experimental condition) were seeded into the upper wells of the chamber and incubated for 6 or 8 h at 37°C in the medium without FBS. Medium with 10% FBS was used as a chemoattractant in the bottom wells. Cells that did not migrate through the pores of the chamber were manually removed with a rubber swab. Cells that migrated to the lower side of the membrane were stained with Eosin and photographed using an inverted microscope.

**Transwell Invasion Assay**

The invasion assay was perfomed using the 24-well transwell (Corning). Upper chamber was coated with 50μl matrigel, and 1.0~5.0x10^5^ (depending on cell type and experimental condition) HCC cells in serum-free medium were added to the upper chamber after matrigel became solid at 37^o^C, and medium with fetal bovine serum was added into bottom wells. Cells were incubated for 48h, then stained with 0.1% Crystal Violet and photographed using an inverted microscope.

**Immunofluorescence Microscopy**

HCC cells were digested with trypsin and plated on cover slides. On the next day, the cells were washed thrice with phosphate buffered saline (PBS) at room temperature, and fixed in 4% formaldehyde on ice for 30 min. Cells were blocked with 2% bovine serum albumin at room temperature for 1h. Primary antibody incubations were performed overnight at 4 ^o^C (PTEN 1:100, CMKLR1 1:100, MMP1 1:200). After extensive washing with PBS, secondary antibody (Alexa Fluor 488-conjugated donkey anti-rabbit/mouse IgG and Alexa Fluor 555-conjugated goat anti-rabbit IgG) incubations were performed at room temperature for 1 h. Samples were washed again with PBS and then mounted in Mowiol 4–88 (Sigma-Aldrich Inc., St Louis, MO, USA). Fluorescence was monitored by inverted confocal laser microscopy (Carl Zeiss, NY, USA).

**Immunoprecipitation**

Cells were washed with ice-cold PBS and lysed in IP lysis buffer (50mM Tris, 150mM NaCl, 1% Triton X-100, 5mM EDTA with Protease Inhibitor Cocktail (Sigma), pH 7.4), incubated on ice for 15min before cellular debris were removed by centrifugation at 14000 rpm for 10min. Supernatant was incubated with antibody overnight at 4^o^C. Protein G Sepharose (GE Healthcare) beads in a 50:50 mixture in 50mM Tris buffer, pH 7.4, were added, and further incubated for another 4 h at 4^o^C. The immunoprecipitates were washed thrice in Tris-buffered saline and boiled for 5min in loading buffer.

**Precipitation of secreted proteins from the tissue culture media**

HCC cells were plated in 100mm dishes. Two days after plating, the cells were washed twice with basal DMEM (serumfree without further additions) and the media was replaced with basal DMEM overnight. The following day, the culture supernatant was collected and centrifuged to pellet any floating cells. Culture supernatants were incubated on ice with 20% trichloroacetic acid (TCA) for 2h on ice to precipitate out all proteins. Precipitated protein was then recovered by centrifugation at 14,000 rpm for 15 min. Protein pellets were washed 5 times with ice cold acetone, briefly allowed to dry, resolved in SDS-PAGE bubffer (without bromophenol blue) by repeated votex, pipetting up and down, freezing and thawing. The dissolved proteins were quantified and the same amount of proteins were analyzed by Western Blot.

**GST Pull-Down Assay**

The full length cDNA fragment of PTEN and CMKLR1 was amplified by PCR and introduced into the pGEX-4T-1 vector to produce GST-PTEN/CMKLR1 proteins in the E.Coli BL21 bacteria with 0.1mM isopropyl β-D-1-thiogalactopyranoside inducement. The fusion protein of was purified using Glutathione-Sepharose 4B (GE Healthcare), as indicated by the manufacturer . Approximately 5 μg GST or GST fusion protein was added to HCC cell lysates, followed by overnight incubation with gentle rotation and then Glutathione-Sepharose 4B were added and incubated for another 3h. Beads were washed three times with cell lysis buffer, and proteins were eluted with 2xSDS loading buffer and analyzed by Western Blot.

**Ubiquitination analysis of PTEN**

HCC cells were treated with 10uM MG132 for 8 hours, cells were harvested, washed with PBS, pellted, and lysed in IP lysis buffer. Equal amounts of cell lysates were incubated with anti-PTEN (mouse) antibody overnight, and then incubated with Protein G Sepharose. The precipitated PTEN proteins were separated by SDS-PAGE and detected by immunoblotting with anti-Ub and anti-PTEN (rabbit) antibodies.

**PTEN lipid phosphatase activity**

Equal amounts of cell lysates were subjected to PTEN immunoprecipitation by the addition of 8ul anti-PTEN mAb, and the immuno-complex formed was captured by incubation with protein G beads for 4h with gentle rotation at 4^o^C. The beads were washed twice in lysis buffer and once in enzyme reaction buffer (ERB) (25mM Tris-HCl, PH 7.4, 140mM NaCl, 2.7mM KCl, and 10mM dithiothreitol[DTT]). The reaction was initiated by adding ERB containing the substrate-dioctanoyl phosphatidylinositol 3,4,5-trisphosphate (PIP3-DiC8) (P-3908; Echelon) to 50μm final concentration; it was left for 4h at 37^o^C and stopped by 100μl malachite green solution (Echlon), and the absorbance was read at 620nm after 15min of incubation at room temperature. A PIP3-only bland was used in parallel to correct for potential nonspecific phosphate release. A standard curve was made by using the phosphate solution provided with the kit.

**Recombinant human chemerin purification**

The full length of human chemerin gene (GeneBank Accession No. NM_002889.3) is 767 base pairs, with a coding sequence (CDS) of 492bp. The first 60bp of CDS encodes a 20-aa signal peptide while the last 21bp encodes a 6-aa peptide which needs to be removed from the immature protein. The fragment of interest was amplified by PCR from hchemerin expression construct using the primers:

F: 5’- GGAATTCCATATGGAGCTCACGGAAGCCCAGCGCCG-3’,

R: 5’- CCGCTCGAGTTAGGAGAAGGCGAACTGTCCAG-3’.

The PCR products were digested with Nde I and Xho I, and cloned into the vector pET-30a (+) (Novagen), which was further identified by sequence. The recombinant pET-30a (+)-hchemerin gene was chemically transformed into the competent E.Coli BL21 bacteria.

The subsequent expression and purification of mature hchemerin was performed mainly following the method described by Xiang et al. [^8^](#_ENREF_8). The protein was concentrated and dialyzed against PBS.

***In vivo* metastasis assay using left ventricular injection model**

Luciferase-expressing PVTT-1 con and PVTT-1 che cells (5.0×10^5^ in 100μl DMEM) were injected into the left ventricles of nude mice, respectively. The metastases were monitored every week. Before mice were anesthetized with isoflurane, an aqueous solution of luciferin (150 mg/kg) was intraperitoneally injected 10 min prior to imaging. The animals were placed into a light-tight chamber of the CCD camera system (Xenogen) and the photons emitted from the luciferase-expressing cells within the animal were quantiﬁed for 1 min using the software program Living Image (Xenogen).

**Chemerin treatment of tumor-bearing nude mice**

The therapeutic potential of chemerin was tested in both left ventricular injection model and intrahepatic injection model, respectively. PVTT-1 luci cells were used in both of the two models. For the left ventricular injection model, the injected mice were divided into two groups randomly, and were intraperitoneally injected with recombinant human chemerin (0.5mg/kg) or equal volume of PBS after recovery (~0.5h after left ventricular injection). The intraperitoneal injection of chemerin or PBS was performed once every other day. The luciferase signals were examined every week, and the death of each mouse was recorded.

For the intrahepatic injection model, the luciferase signals were examined 3 days after the operation, and the mice showing positive signals (~95-100% of the injected mice) were divided into two groups at random, and were intraperitoneally injected of recombinant human chemerin (0.5mg/kg) or equal volume of PBS every other day. The weight and death of each mouse were recorded for the drawing of weight curve and survival curve, respectively. The superficial hepatic foci were counted at the end of the experiment and statistical analyzed.

**References for Supplementary Materials and Methods**

1. Qin XF, An DS, Chen IS and Baltimore D. Inhibiting HIV-1 infection in human T cells by lentiviral-mediated delivery of small interfering RNA against CCR5. *Proc Natl Acad Sci U S A* 2003; **100**: 183-188.

2. Li G, Ji XD, Gao H, Zhao JS, Xu JF, Sun ZJ et al. EphB3 suppresses non-small-cell lung cancer metastasis via a PP2A/RACK1/Akt signalling complex. *Nat Commun* 2012; **3**: 667.

3. Wang Y, Liu DP, Chen PP, Koeffler HP, Tong XJ and Xie D. Involvement of IFN regulatory factor (IRF)-1 and IRF-2 in the formation and progression of human esophageal cancers. *Cancer Res* 2007; **67**: 2535-2543.

4. Li Y, Tang ZY, Ye SL, Liu YK, Chen J, Xue Q et al. Establishment of cell clones with different metastatic potential from the metastatic hepatocellular carcinoma cell line MHCC97. *World J Gastroenterol* 2001; **7**: 630-636.

5. Li Y, Tang Y, Ye L, Liu B, Liu K, Chen J et al. Establishment of a hepatocellular carcinoma cell line with unique metastatic characteristics through in vivo selection and screening for metastasis-related genes through cDNA microarray. *J Cancer Res Clin Oncol* 2003; **129**: 43-51.

6. Tian J, Tang ZY, Ye SL, Liu YK, Lin ZY, Chen J et al. New human hepatocellular carcinoma (HCC) cell line with highly metastatic potential (MHCC97) and its expressions of the factors associated with metastasis. *Br J Cancer* 1999; **81**: 814-821.

7. Wang T, Hu HS, Feng YX, Shi J, Li N, Guo WX et al. Characterisation of a novel cell line (CSQT-2) with high metastatic activity derived from portal vein tumour thrombus of hepatocellular carcinoma. *Br J Cancer* 2010; **102**: 1618-1626.

8. Xiang D, Zhang J, Chen Y, Guo Y, Schalow A, Zhang Z et al. Expressions and purification of a mature form of recombinant human Chemerin in Escherichia coli. *Protein Expr Purif* 2010; **69**: 153-158.
