# Supplementary figures and images for: Chemerin suppresses hepatocellular carcinoma metastasis through CMKLR1-PTEN-Akt axis
Source: Br J Cancer. 2018 May 2;118(10):1337–48. doi: 10.1038/s41416-018-0077-y (PMC5959946; doi:10.1038/s41416-018-0077-y)

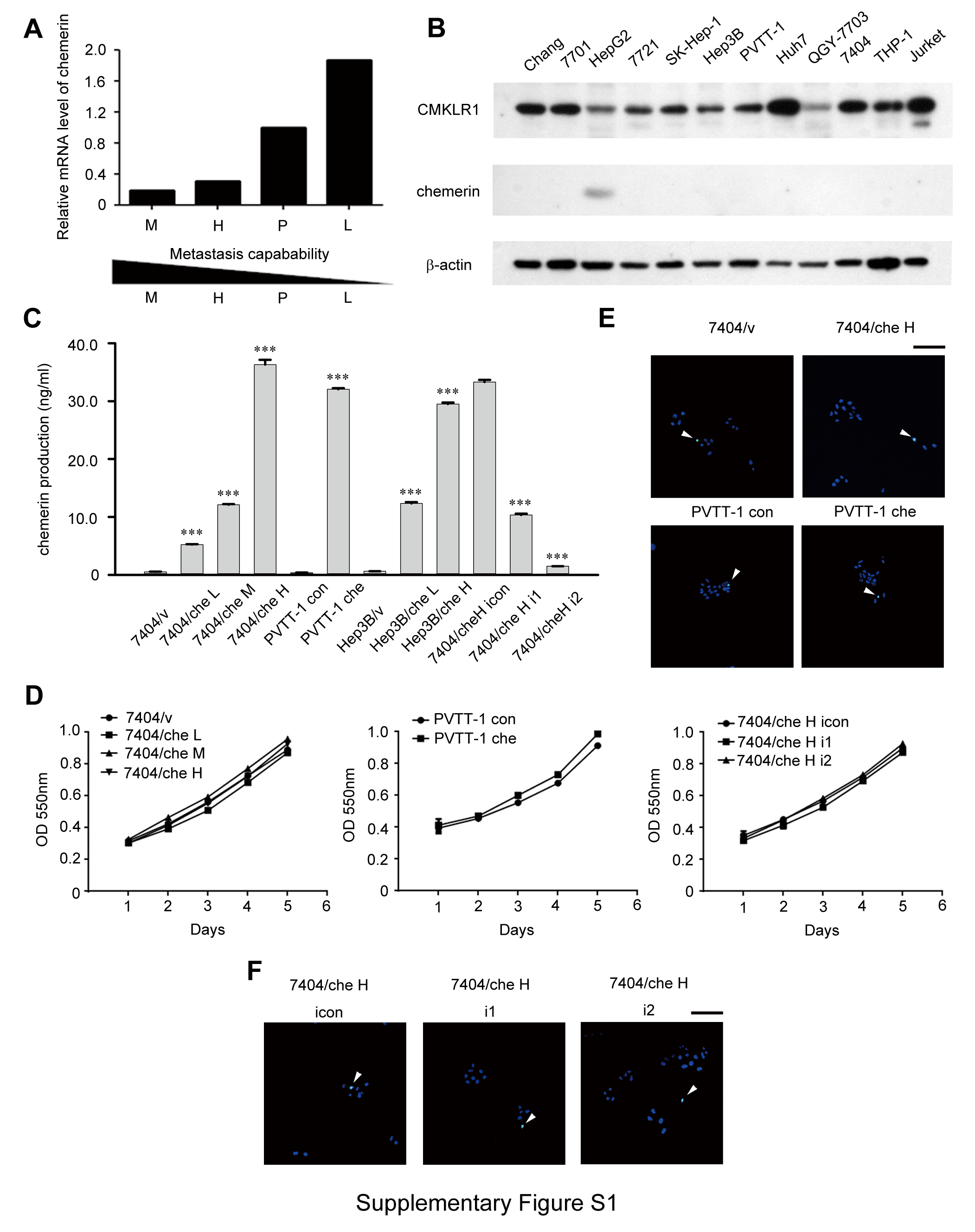

Supplement: Supplementary file 3 — Supplementary Figure 1 [file 41416_2018_77_MOESM3_ESM.tif]

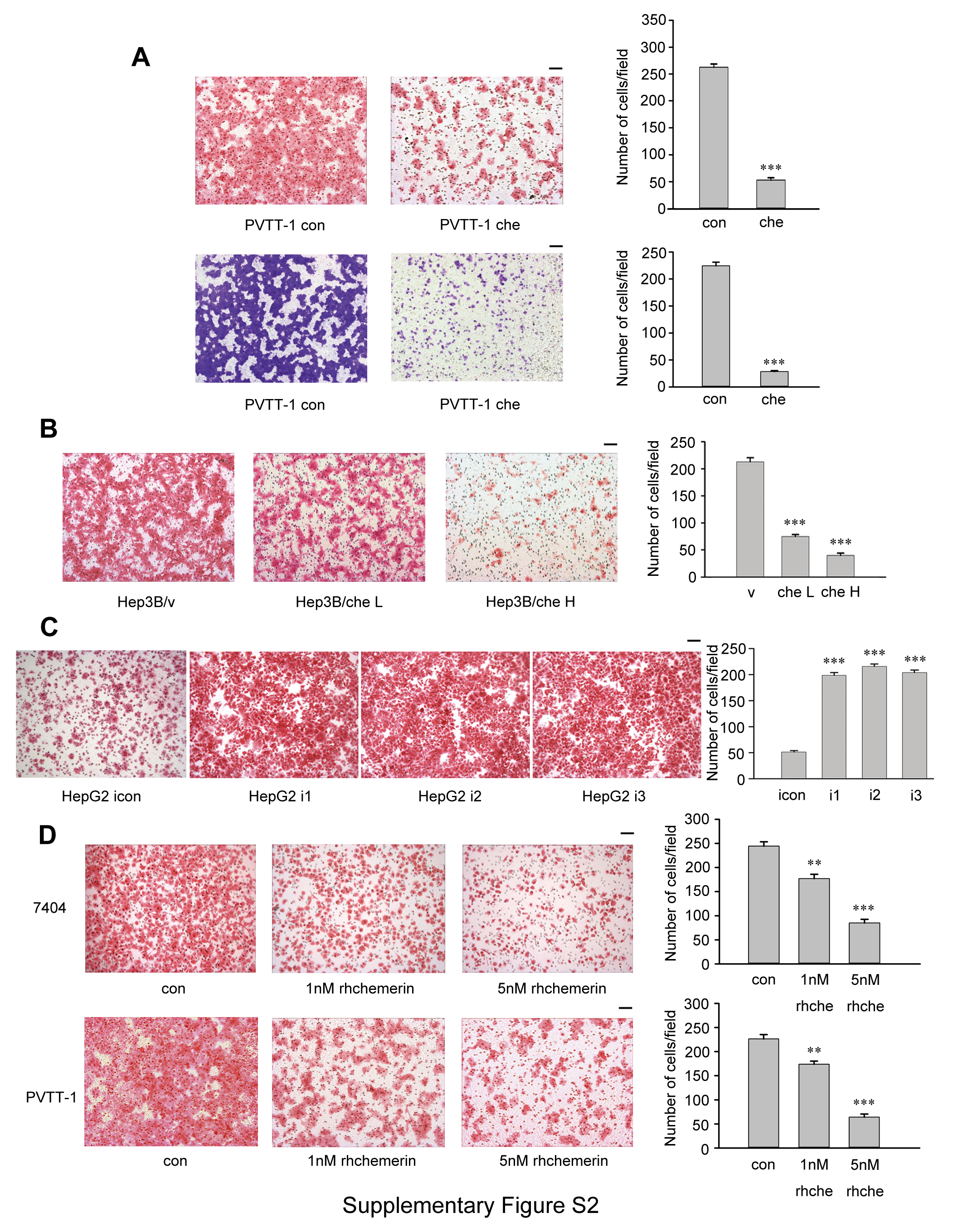

Supplement: Supplementary file 4 — Supplementary Figure 2 [file 41416_2018_77_MOESM4_ESM.tif]

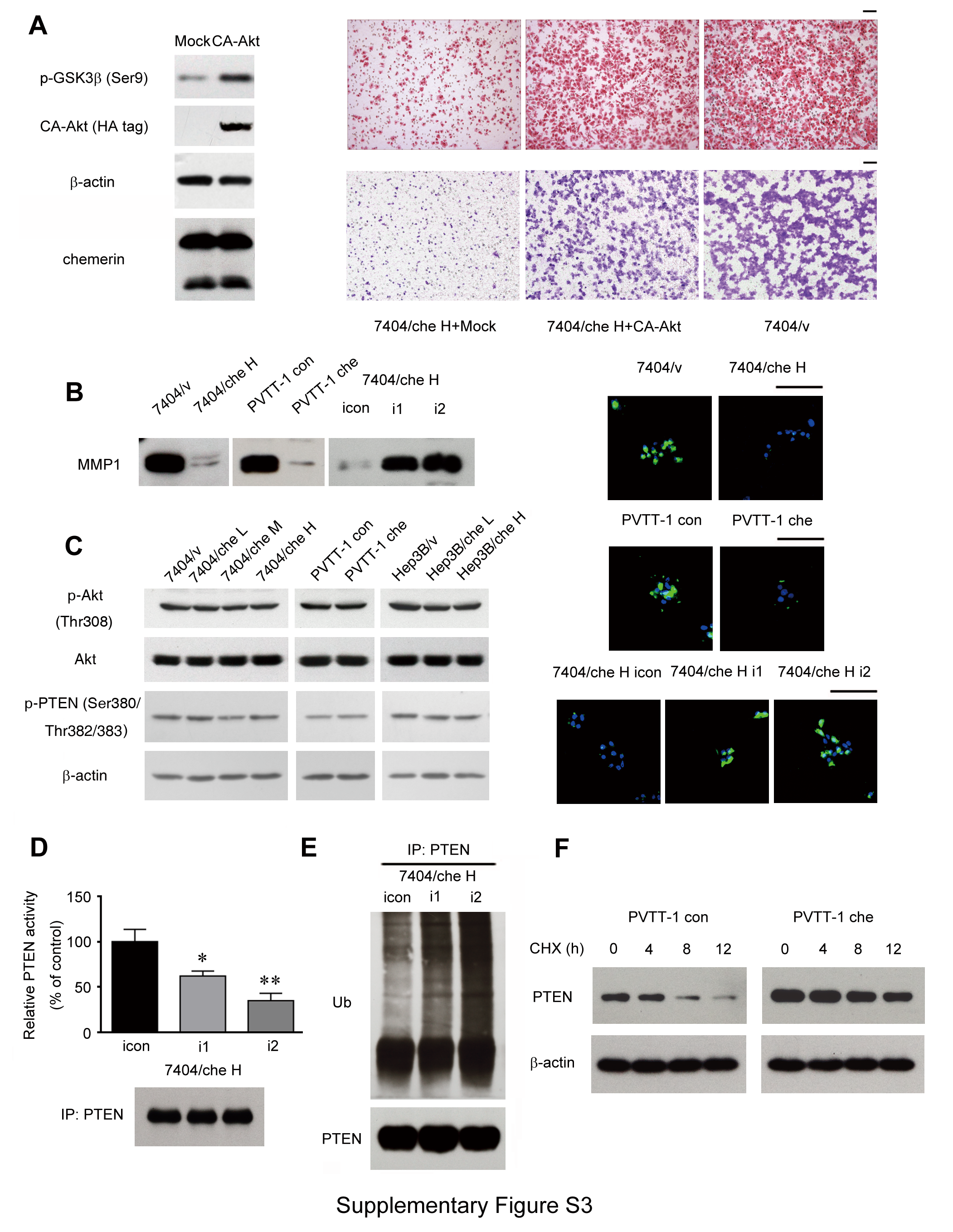

Supplement: Supplementary file 5 — Supplementary Figure 3 [file 41416_2018_77_MOESM5_ESM.tif]

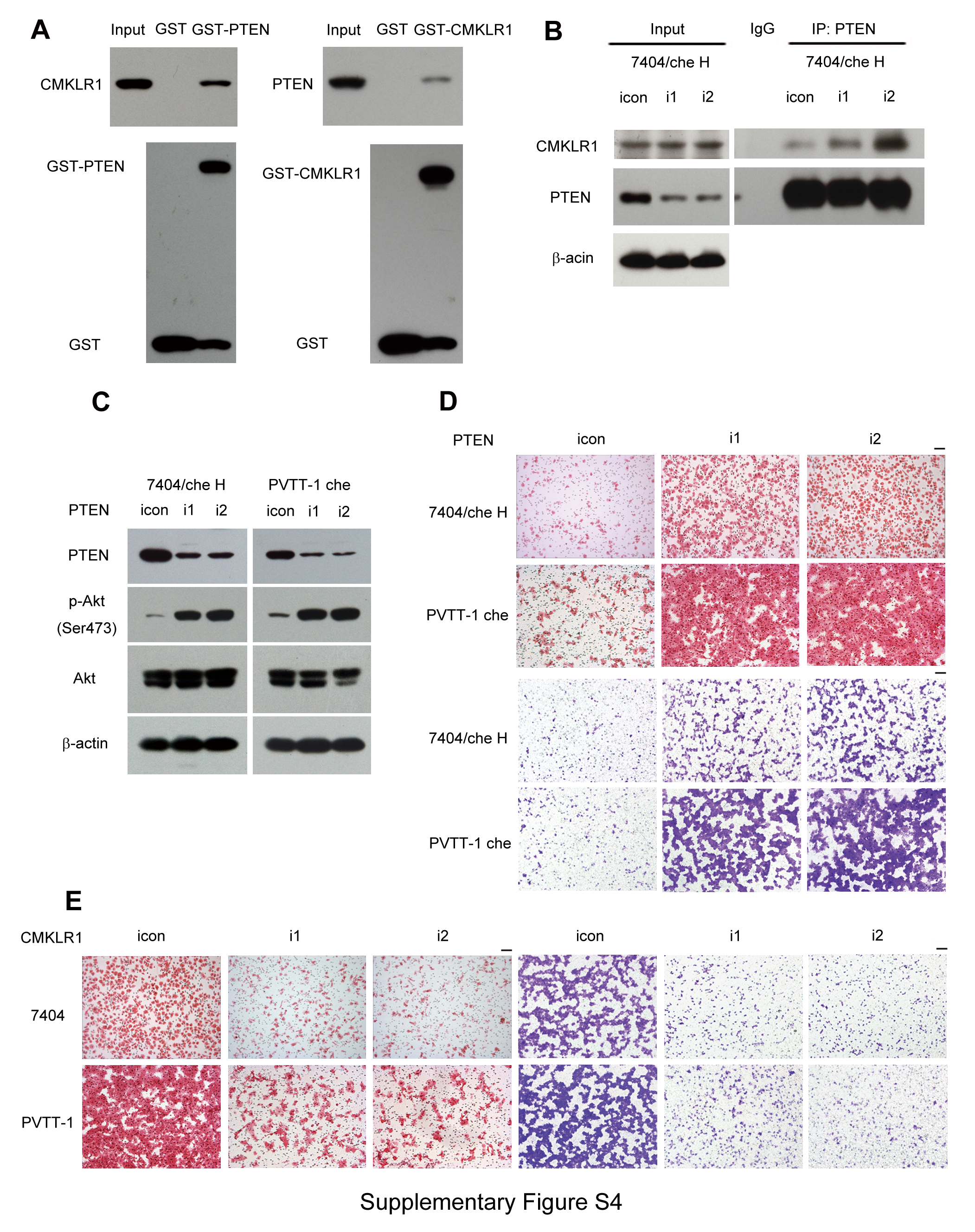

Supplement: Supplementary file 6 — Supplementary Figure 4 [file 41416_2018_77_MOESM6_ESM.tif]

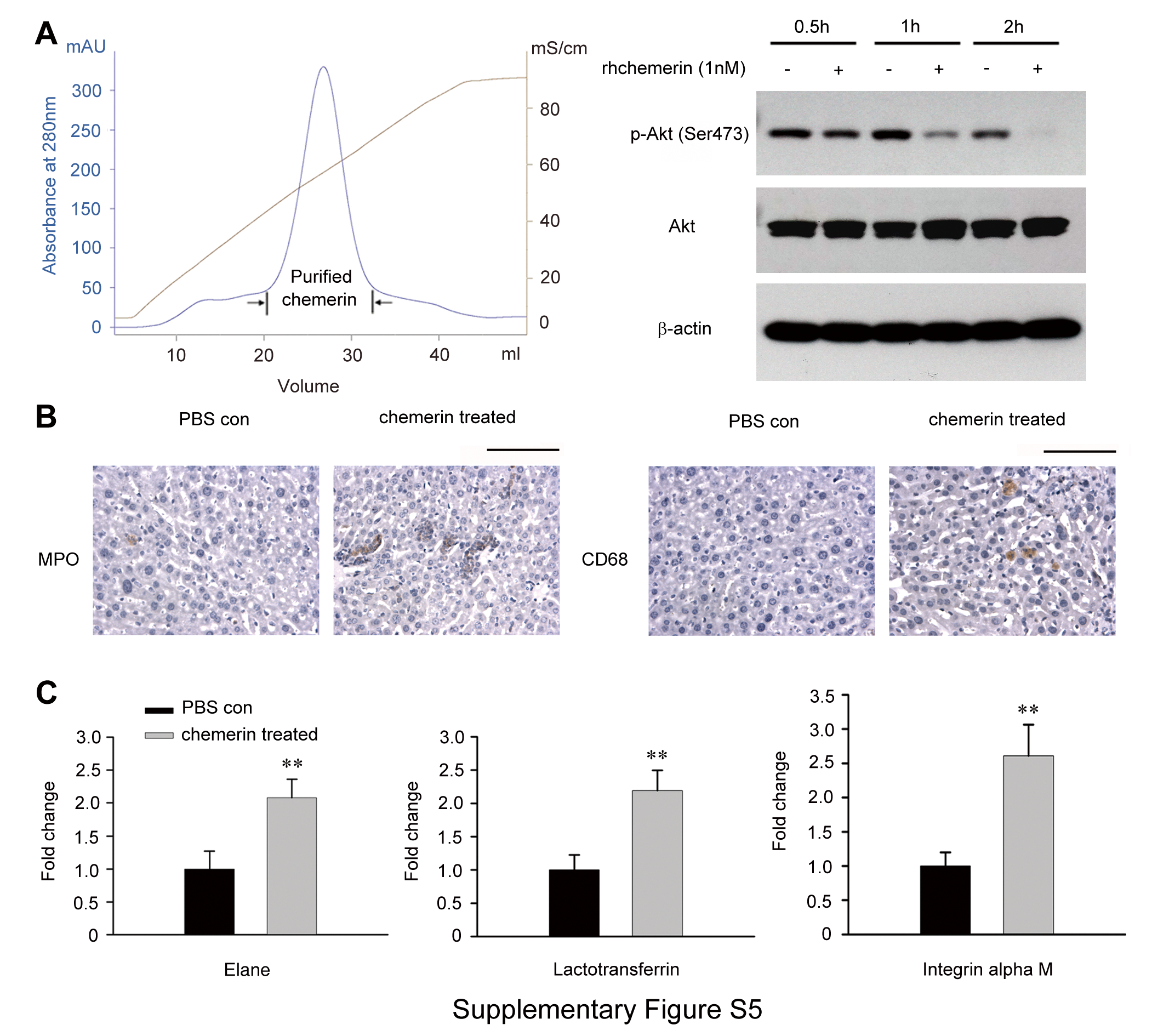

Supplement: Supplementary file 7 — Supplementary Figure 5 [file 41416_2018_77_MOESM7_ESM.tif]
